# A Multicomponent Polymer-Metal-Enzyme System as Electrochemical Biosensor for H_2_O_2_ Detection

**DOI:** 10.3389/fchem.2022.874965

**Published:** 2022-04-29

**Authors:** Pengfei Tong, Muhammad Asif, Muhammad Ajmal, Ayesha Aziz, Yimin Sun

**Affiliations:** ^1^ Henan Institute of Microsurgery, The First Affiliated Hospital, College of Clinical Medicine of Henan University of Science and Technology, Luoyang, China; ^2^ School of Chemistry and Chemical Engineering, Huazhong University of Science and Technology, Wuhan, China; ^3^ Department of Chemistry, College of Chemistry and Chemical Engineering, Xiamen University, Xiamen, China; ^4^ Hubei Key Laboratory of Plasma Chemistry and Advanced Materials, School of Materials Science and Engineering, Wuhan Institute of Technology, Wuhan, China

**Keywords:** graphene, multicomponent nanohybrid, electrochemical biosensor, H_2_O_2_ detection, multicomponenet polymer

## Abstract

Herein, an Au nanoparticles-polydopamine-poly acrylic acid-graphene (Au NPs-PDA-PAA-graphene) multicomponent nanohybrid is fabricated by surface functionalization of graphene alongside extensive *in-situ* growth of Au nanoparticles. The as-obtained nanocomposite possesses good hydrophilicity, excellent biocompatibility and high biomolecules loading capacity, which acts as an ideal platform for enzyme modification. Considering this fact, Horseradish peroxidase is expressively immobilized upon Au NPs-PDA-PAA-graphene surface, in order to lay the foundations of a biosensor that is majorly based on enzymatic activity. The biosensor exhibits higher sensitivity towards the determination of H_2_O_2_ with linearity ranging from 0.1 μm upto 20 mm, and the limit of detection going down to 0.02 μm. Encouraged by its acceptable electrocatalytic performance, this multicomponent system can also be easily employed for carrying out the real-time tracking of H_2_O_2_ coming out of Macrophage cells. Therefore, this work designs an extraordinarily updated platform for biosensing related applications, and also presents a reliable platform for the direct detection of H_2_O_2_ *in vivo* and *in vitro*, which show great potential in bioelectroanalytical chemistry, cellular biology, and pathophysiology.

## Introduction

The structural shape of graphene based nanocomposites incorporated with polymers as well as metallic nanoparticles, and other species to harvest synergetic effects and integrated properties of the individuals, has claimed great interest due to their wide range of scientific as well as industrial implications (Badhulika et al., 2015; Sun et al., 2016; Liao et al., 2018; White et al., 2019; Gao et al., 2021). Graphene has, for a long time, been an attractive choice as the building block for nanocomposites owing to its extraordinary mechanical, electronic, and thermal characteristics, as well as the large surface area (Sun et al., 2015; Zeng et al., 2019; Zhao et al., 2020). Graphene nanocomposites with well-designed nanostructures have been extensively fabricated in order to produce diverse materials with desired performance (Aziz et al., 2019a; Asif et al., 2019; Ibrahim et al., 2021). In particular, graphene and metal based nanoparticle composites have been an area of immense significance from the present day research perspective taking advantage of their unmatched implications in the area of sensors, fuel cells, and energy conversions (Nan et al., 2018; Darabdhara et al., 2019). So far, huge progress is reported to have been undertaken for the improved synthesis as well as manufacturing of graphene and metal based nanoparticle composites (Huang et al., 2011; Mangadlao et al., 2017; Ashraf et al., 2020). Among them, graphene-gold nanoparticles (Au NPs) are the ones being investigated the most, and many systematic approaches are reported to prepare Au NPs with various sizes (Asif et al., 2018a; Li et al., 2021). However, some challenges still remain in those methods, such as how to prevent the aggregation and restack of graphene sheet to get higher specific surface areas; how to ensure improved biocompatibility; and how to keep in check the spatial distribution as well as size for the nanoparticles on the surface of graphene. To address those problems, a surface modification strategy of graphene is proposed to fabricate an efficient substrate for the development and growth of gold NPs.

Covalent binding of polymers with graphene through interfacial interactions represents an effective approach for the development of special functional graphene composite (Sun et al., 2020; Perumal et al., 2021). When incorporated with polymer, the aggregation of graphene nanosheets will be effectively inhibited, thus providing the large surface area (Sun et al., 2017). In this paper, covalently functionalized graphene sheets are prepared through grafting poly acrylic acid (PAA) chains on its surface. PAA is a water soluble polymer with many advantages, such as good biocompatibility, non-toxicity, and easy chemical modification (Kausar, 2020). Both hydrophilicity and biocompatibility of graphene could be realized by functionalization of graphene with PAA. Because of plethora carbonyl groups on PAA, dopamine (DA) can be covalently bonded to PAA chain to form PDA-PAA-graphene. Dopamine, a naturally occurring neurotransmitter, found in a variety of animals, is generally regarded as a unique component that can mimick the proteins which are adhesive in nature (Li et al., 2015). Moreover, DA is also used as a reducing agent and stabilizer which is commonly used for the synthesis of metal-nanoparticles (Au, Ag, Pt et al.) by directly reducing the salt of metal precursor (Zhang et al., 2013; Ren et al., 2016; Wen et al., 2018). By using the *in situ* reducing method, Au NPs could easily be distributed upon the surface of the graphene nanosheets in a homogeneous way. The resulting Au NPs-PDA-PAA-graphene nanocomposites work an ideal platform for the modification of biomolecules. Horseradish peroxidase (HRP), with this unique ability of catalyzing the oxidation for an ample amount of substrates *via* hydrogen peroxide or any other closely related components, is hailed as a mainstream tool metalloenzyme for establishing the electrochemical biosensors (Sellami et al., 2022). As a potential application, by further linking peroxidase, an amplified biosensing toward H_2_O_2_ at such Au NPs-PDA-PAA-graphene nanocomposites is constructed.

H_2_O_2_ is reckoned as an extremely stable reactive oxygen species, which serves as a transporter in a variety of ongoing cellular activities, which holds a pivotal contribution in the case of dealing with unwanted oxidative stress, slowing down the aging process, as well as combating the pathological processes, like cancer, neurological disarray, and cardiovascular issues (Xiao et al., 2012; Asif et al., 2017; Zhang et al., 2017; Asif et al., 2018b; Aziz et al., 2019b; Zhao et al., 2021). Therefore, the quantitative determination of intracellular and extracellular H_2_O_2_ is known to offer more comprehensive illustration of the clinical aspects of the rise in H_2_O_2_ concentrations, alongside its assistance in studies put forward to elaborate the biological implications of H_2_O_2_ in cells (Xi et al., 2018; Ma et al., 2020; Yuan et al., 2020; Zhang et al., 2020; Wang et al., 2021; Zong et al., 2021).

In this paper, a novel Au NPs-PDA-PAA-graphene composite film is designed, which offers superior results both in loading and in the activity of immobilized enzymes. Apart from this, the mentioned film also presents improved electrocatalysis/nano-enhancement activity for H_2_O_2_ biosensing. PAA as a “friendly” and “soft” linker, DA as a coupling and reducing agent for HAuCl_4_, Au NPs as a catalyst for electrode reaction, and HRP as an enzyme, are mixed to yield graphene-based bionanocomposites. The proposed material showcases impressive biocompatibility, superbly uniform distribution of the nanoparticles, and a much higher loading/activity of the immobilized enzymes. This nanohybrid system shows immensely bettered amperometric biosensing activity in contrast to those based on traditional methods and techniques, e.g., a higher detection sensitivity or a sub-micromolar level detection limit, which makes H_2_O_2_ extremely useful for both *in vivo* and *in vitro detections.* This graphene-based bionanocomposites enzyme electrode shows great potential application in the second-generation style of biosensing.

## Materials and Methods

### Preparation of Au NPs-PDA-PAA-Graphene

The preparation of PAA-graphene: The synthesis of PAA-graphene was performed by hydrolyzing poly tert-butylacrylate (PtBA). The synthetic procedure of PtBA-graphene was described in our previously published paper (Sun et al., 2018). Brifly, 200 mg graphene-PtBA was distributed into 20 ml CH_2_Cl_2_, and 1 ml trifluoroacetic acid (TFA) was added. Later, the prepared mixture was stirred at 40°C for 36 h. Then, CH_2_Cl_2_ was removed by centrifugation, and sediment was washed thoroughly by acetone and water to get pure PAA-graphene.

The synthesis of PDA-PAA-graphene: PAA-graphene (200 mg) was dissolved in 10 ml of deionized water, Na_2_CO_3_ solution was poured to adjust the pH to 8.5. After sonication for 0.5 h, the solution became homogeneous. This solution was then transferred to an ice-bath, and followed by the addition of N-Hydroxysuccinimide (NHS, 50 mg) and 1-(3-Dimethylaminopropyl)-3-ethylcarbodiimide hydrochloride (EDCHCl, 200 mg), the mixture solution (denoted as solution A) was made to stir under N_2_ for about 15 min. Meanwhile, Na_2_CO_3_ (55 mg) was dissolved in 10 ml of deionized water in order to prepare solution B. This solution was also made to stir under N_2_ for about 15 min, and then dopamine HCl (240 mg) was poured in with yet another degassing for about 15 min. Later, solution B was mixed with solution A and was again stirred for about 30 min inside an iced-water bath for about 12 h at around 35°C. During this process, dopamine is easy to polymerize to form polydopamine in alkaline conditions. Eventually, the reaction mixture was made to centrifuge and was also washed thoroughly using deionized water to achieve PDA-PAA-graphene.

The preparation of Au NPs-PDA-PAA-graphene: PDA-PAA-graphene (10.0 mg) was put inside a reaction vessel, and was poured with 10 ml of HAuCl_4_ solution (50 mm). The reaction was allowed to continue overnight at the expected room temperature. At the end of the reaction, the Au NPs deposited PDA-PAA-graphene was removed from the suspension *via* centrifugation process and were frequently washed with water.

### The Preparation of Enzyme Electrode

The enzyme based electrode was designed by immersing Au NPs-PDA-PAA-graphene electrodes into phosphate buffer solution (PBS) with 0.2 mg ml^−1^ HRP for overnight reaction, followed by washing with Milli-Q water and then drying at 4°C.

### Characterization

A Kratos-Axis spectrometer having monochromatic Al KR of (1,486.71 eV), an X-ray radiation of (15 kV and 10 mA) and a strong hemispherical electron energy analyzer, was used to carry out X-ray photoelectron spectroscopy (XPS) determinations. Casa XPS software was employed to accomplish curve fitting and background suppression. Fourier-transformed infrared spectrum (FT-IR) was observed on Perkin Elmer FTIR. Thermogravimetric Analysis (TGA) was achieved with the help of TA Instruments TGA/DSC. 1H NMR measurement was performed on a Bruker AVANCE NEO 400 M equipment with D_2_O as solvent. UV-vis spectra were observed on a Schimadu UV-2550 spectrometer. Scanning electron microscopy images were acquired by using FESEM instrument (JSM-6700F, Japan). CV and chronoamperometric analysis were undertaken by a CHI 660 D electrochemical workstation (CH Instrument Company). For this purpose, a traditional three-electrode system was selected. HRP/Au NPs-PDA-PAA-graphene electrode was used as a working electrode. The auxiliary and reference electrodes were Pt wire and Ag/AgCl, respectively. All the measurements were conducted at room temperature.

### Cell Culture

ATCC (United States) supplied the macrophages and the cells containing Bovine Brain Extract (BBE) supplement were stored in Endothelial Cell Growth Medium (EGM). EGM^®^ is a modified MCDB 131 formulation and is supplemented with 10 μg/ml hEGF (human recombinant Epidermal Growth Factor), 1.0 mg/ml Hydrocortisone, 50 mg/ml Gentamicin, 50 μg/ml Amphotericin B, 3 mg/ml Bovine Brain Extract (2 ml), 2% v/v Fetal Bovine Serum (FBS) and stored at 37°C in humid conditions with 5% CO_2_. 24-well plate was used to seed macrophages where they gained 80% confluency before experiments. Calcein-AM was used for living cell staining. Macrophages cells were incubated at 37°C for 15 min before imaging.

## Results and Discussion

**FIGURE 1:**
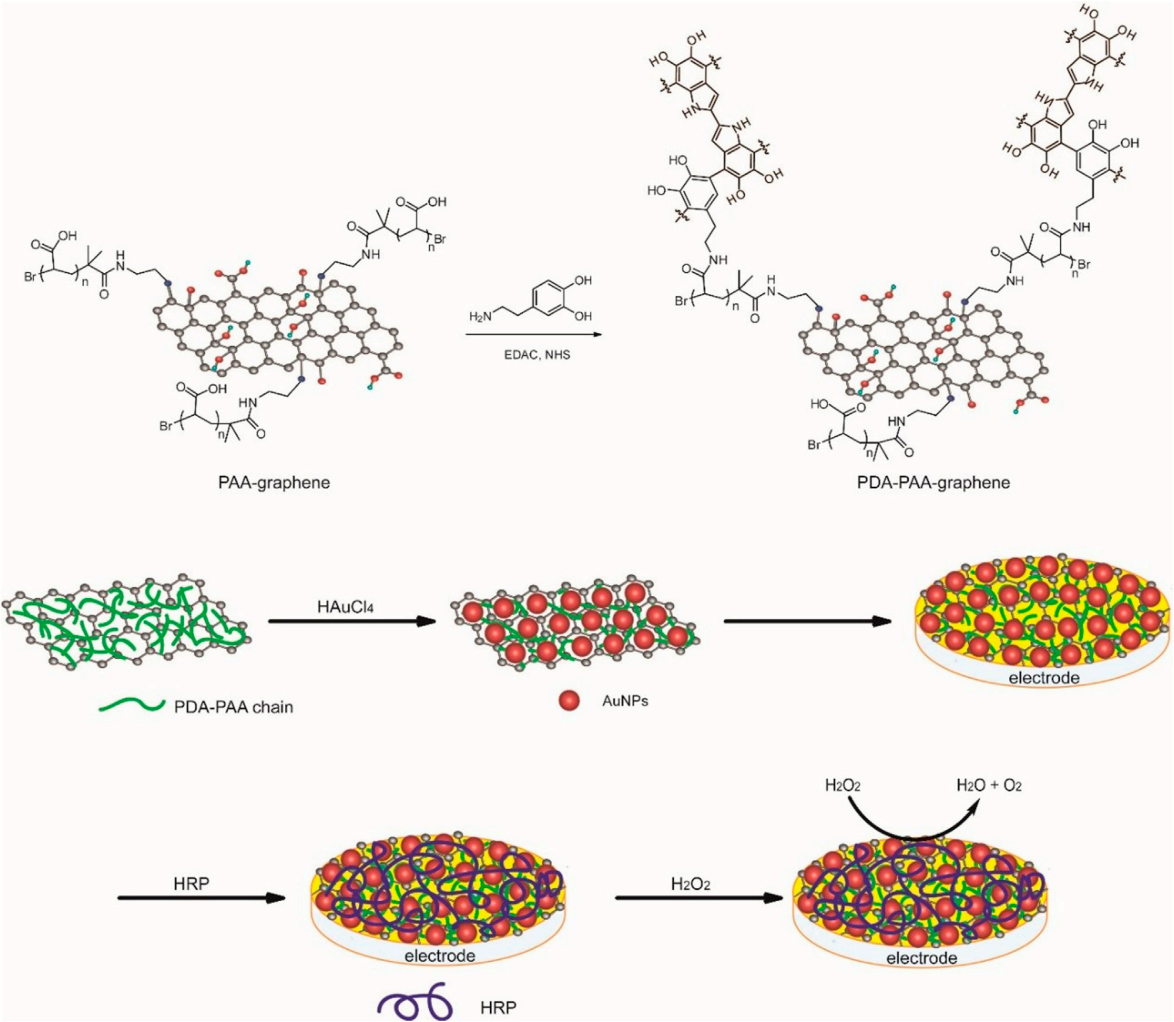
The preparation of the Au NPs-PDA-PAA-graphene composite and the construction of enzyme electrode.

The detailed process employed for carrying out the preparation of the Au NPs-PDA-PAA-graphene composite is illustrated in Figure 1. The synthetic procedure of PAA-graphene was reported previously (Sun et al., 2018), then DA is covalent linked to PAA *via* the condensation reaction between the NH_2_ group in DA and the carboxyl group in PAA, meanwhile, DA will polymerize to form polydopamine (PDA) in alkaline conditions. Next, Au NPs are immobilized onto PDA-PAA-graphene by *in situ* reducing of HAuCl_4_ by dopamine. Au NPs-PDA-PAA-graphene was coated on the glassy carbon electrode to build a platform for enzyme loading. Finally, HRP was absorbed on the electrode to construct an enzyme electrode.

**FIGURE 2:**
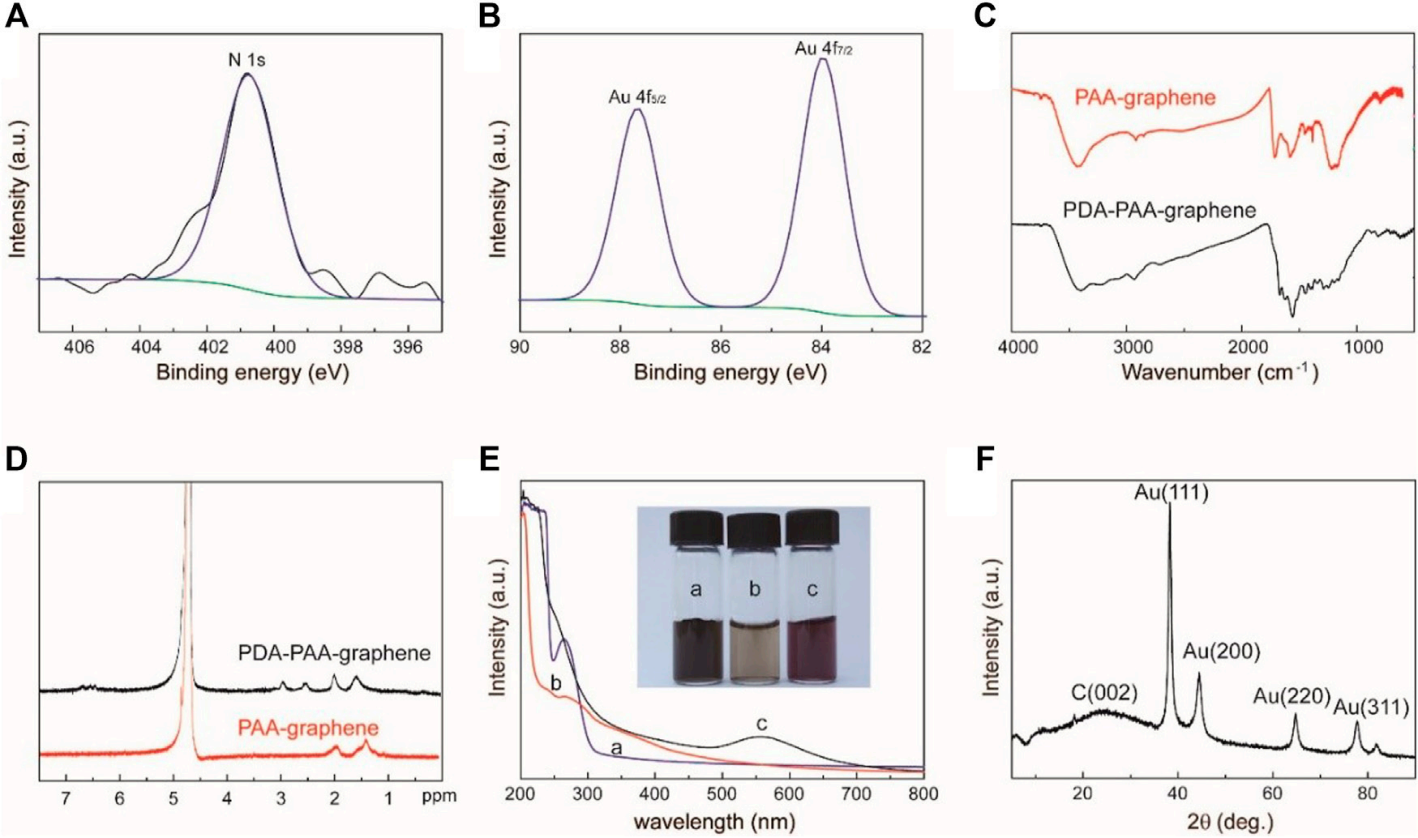
**(A)** The N1s XPS spectra of Au NPs-PDA-PAA-graphene **(B)** The Au4f XPS spectra of Au NPs-PDA-PAA-graphene **(C)** FT-IR spectra for PAA-graphene and PDA-PAA-graphene) **(D)** 1H NMR spectrum of PDA-PAA-graphene and PAA-graphene in D_2_O **(E)** UV-vis absorption spectra and photographs (inset) of PAA-graphene **(A)**, PDA-PAA-graphene **(B)** and Au NPs-PDA-PAA-graphene **(C)** solutions **(F)** XRD pattern of Au NPs-PDA-PAA-graphene composite.

X-ray photoelectron spectroscopy (XPS) is first applied to trace the synthesis process. The condensation reaction between dopamine and PAA-graphene can be verified by noticing a new absorbance peak at 400.8 eV linked to the binding energy of N1s in an XPS spectrum of PDA-PAA-graphene (Figure 2A). After introducing Au NPs to PDA-PAA-graphene, the representing peaks of Au 4f_7/2_ and Au 4f_5/2_ show up at the binding energies of 83.9 and 87.7 eV respectively (Figure 2B), suggesting the successful immobilization of Au NPs upon the PDA-PAA-graphene surface. FT-IR measurement provides further evidences of the above chemical changes as the carboxylate peak in PAA is no longer present, and amine and aromatic rings peaks appear at 1,670, 1,620, and 1,550 cm^−1^, individually (Figure 2C) (Wu et al., 2011).

The condensation reaction can also be identified by 1H NMR spectrum (Figure 2D). Comparing with that of PAA-graphene, new peaks appear at *d* 6.83–6.40 and 2.5–3.0 at the 1H NMR spectrum of PDA-PAA-graphene, which directly corresponds to the chemical shift of protons in aromatic rings and alkyl of DA. f = A/A_0_ is used to determine the dopamine content (mol%) in PDA-PAA-graphene. Where, A stands for the integral part of the peaks at *d* 6.83–6.40 representing the quantity of H in the aromatic rings of the given catechol moieties, while A_0_ corresponds to the integral part of the peaks at *d* 2.2–1.2 showcasing the quantity of H in the PAA backbone. The dopamine ratio found in the resultant polymer is nearly 25% as per the given method. This result is a little lower than that calculated by thermogravimetric analysis (31%) (Supplementary Figure S1).

The as-prepared Au NPs-PDA-PAA-graphene composite is also confirmed by UV-vis absorption spectrum as displayed in Figure 2E. The spectrum of PAA-graphene presents a maximum absorption at 263 nm, which signifies π-π* transitions between the aromatic C=C bonds (He et al., 2011). After reacting with DA, a shoulder peak at 275 nm, a characteristic absorption for catechol, is detected (Bui et al., 2021). After reduction of HAuCl_4_, a relatively new peak at 556 nm appears and the solution becomes violet black in color. This newly appearing peak can well be stemming from the surface plasmon resonance (SPR) absorptive band for Au NPs, highlighting the reducibility of AuCl_4_^−1^ to Au NPs in a successful way (Mawlud et al., 2017). In contrast to the as-prepared Au colloids present in aqueous solution (Song et al., 2011), the obtained absorption maxima is found to be red-shifted, which in turn can consequently be attributed to the altering refractive index and to some extent partial aggregation of the seed particles inside the growth solution. These results further go on to prove that as far as *in situ* methods are concerned, Au nanoparticles are closely linked with each other for the sake of the reduction process and also to small quantities of agglomerations or even bigger particles are expected to have deposited on the graphene surface. The crystalline XRD pattern of Au NPs-PDA-PAA-graphene nanohybrid (Figure 2F) show a peak at 24.3 indexed to the (002) crystal face of graphene, and the peaks at 38.1, 44.3, 64.5, and 77.6 correspond to the (111) (200), (220), and 311) crystal faces of Au nanoparticles (JCPDS 4–0,783) (Li et al., 2012). Quantitative measurements of Au NPs on Au NPs-PDA-PAA-graphene surface are materialized by EDX, pointing out the presence of 58 wt% of Au NPs (Supplementary Figure S2).

**FIGURE 3:**
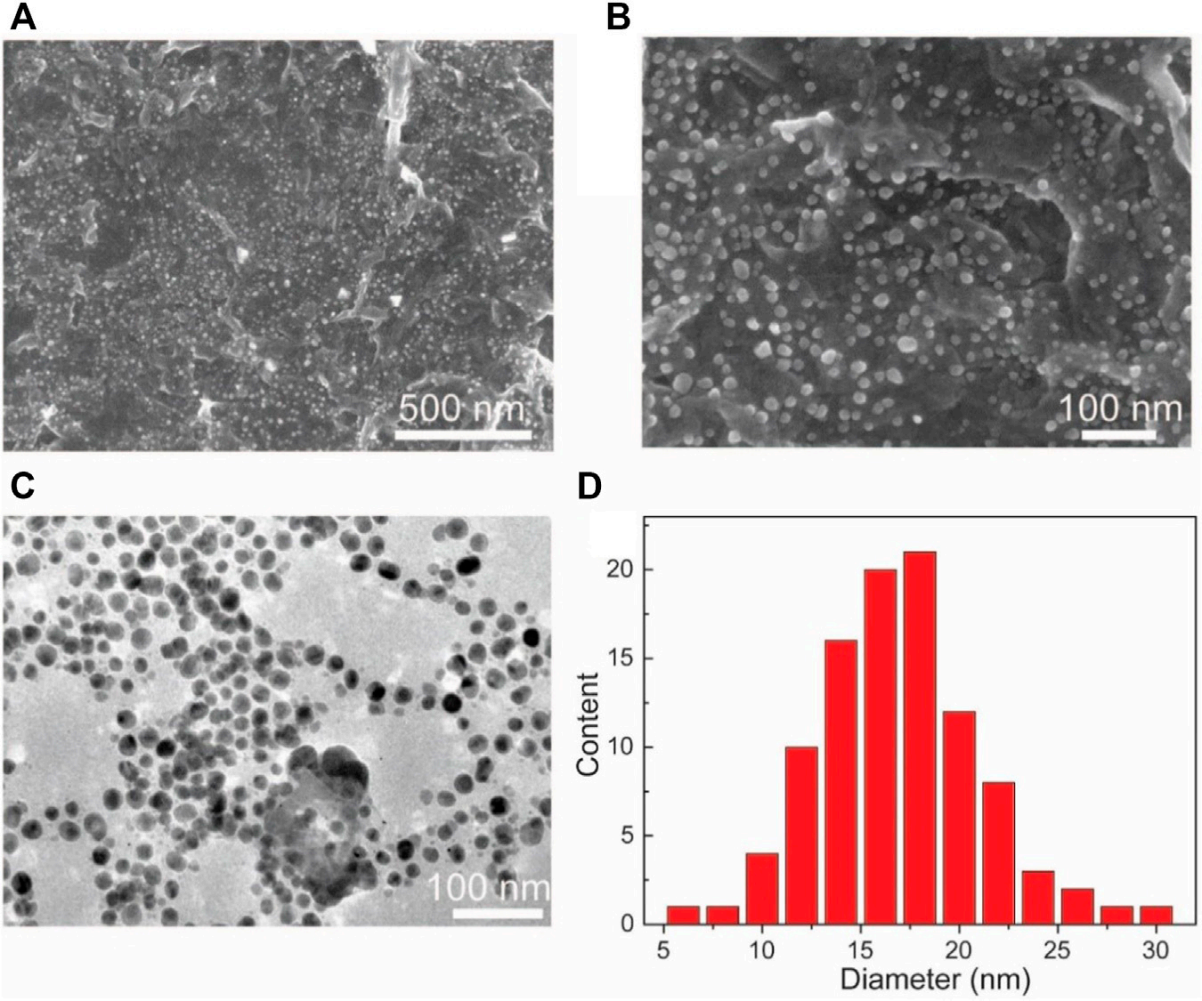
**(A,B)** SEM based images of Au NPs-PDA-PAA-graphene with varying magnification **(C)** TEM image of Au NPs-PDA-PAA-graphene **(D)** Size and distribution of Au nanoparticles in statistics.

SEM and TEM are used to undertake the characterization of the morphology of the Au NPs-PDA-PAA-graphene composites. Figures 3A,B, represent the random distribution of Au NPs PDA-PAA-graphene surface. These also show the fact that the nanoparticles are well embedded on the interior of the surface, highlighting a close interaction among the NPs and PDA-PAA modified graphene sheets. The distribution of Au NPs is believed to be relatively uniform with no severe aggregations. TEM images signify samples to be composed of nanoparticles having diameters averaging nearly 18 nm and the size distribution ranging between 5 and 30 nm. The diameter of most of the found particles is between 12 and 20 nm (Figures 3C,D).

**FIGURE 4:**
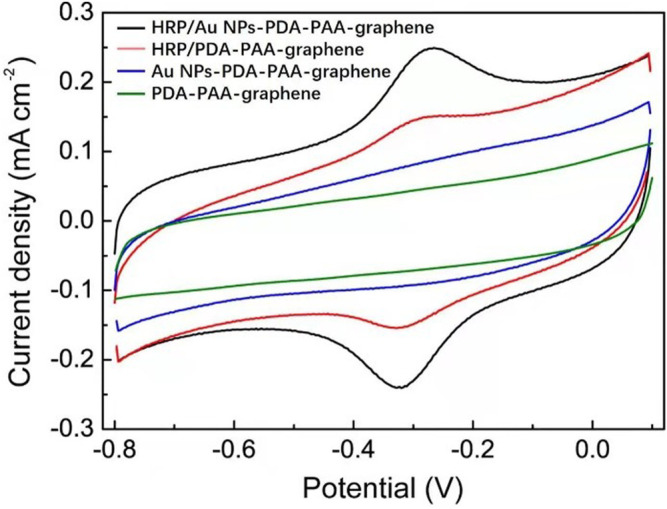
CV curves of HRP/Au NPs-PDA-PAA-graphene, HRP/PDA-PAA-graphene, Au NPs-PDA-PAA-graphene and PDA-PAA-graphene in 0.1 M PBS (pH 7.4) having 1 mM H_2_O_2_ at the Scan rate of (50 mV s^−1^).

The novel nanostructure of the Au NPs-PDA-PAA-graphene inspires us to investigate it as enzyme biosensor. Figure 4 is the CV representation of differently modified electrodes in 50 mm PBS (pH = 7.4), with 50 mV s^−1^ scan rate. In the CVs of Au NPs-PDA-PAA-graphene and PDA-PAA-graphene electrodes, no observable redox peak appeared which is indicative of their electrochemical inactivity at the specific potential range (−0.8 + 0.1 V). A clearly distinguishable couple of redox peaks in the CVs of HRP/PDA-PAA-graphene are attributed to the Fe^III^/Fe^II^ redox coupling related to the heme protein for HRP (Liu et al., 2012). While in the CV curve of HRP/Au NPs-PDA-PAA-graphene, the Fe^III^/Fe^II^ redox couple is more distinct with the cathodic peak potential (−0.320 V) and anodic peak potentials (−0.267 V). Considering the conventional potential [E_o’_ = (Epa + Epc)/2] of −0.294 V, and peak point separation (ΔEp) ∼53 mV at the scan rate of (50 mV/s), indicating a reversible electrochemical behavior of HRP, which is much better than other HRP-based biosensors known so far (Zong et al., 2006; Tang et al., 2008). This phenomenon is the proof of uninterrupted transfer of electrons between HRP and GCE, initiated by Au NPs as a promoter. The chemistry behind the direct electron transportation between the HRP and GCE is based on the Au NPs, which not only reduce the space for the center of activation for the immobilized HRP and the GCE, but also effectively boost up the electrochemical conductance of the fabricated electrode, in turn, encourages direct electron transportation between the HRP and glassy carbon electrode. As the active sites of HRP mostly buried deep inside the center and make it harder to exchange electrons with the electrode surface (Li et al., 2011), so Au NPs act as a mediator to carry out the fast electron transfer rate which in turn greatly enhanced the electrochemical performance of the synthesized electrode.

**FIGURE 5:**
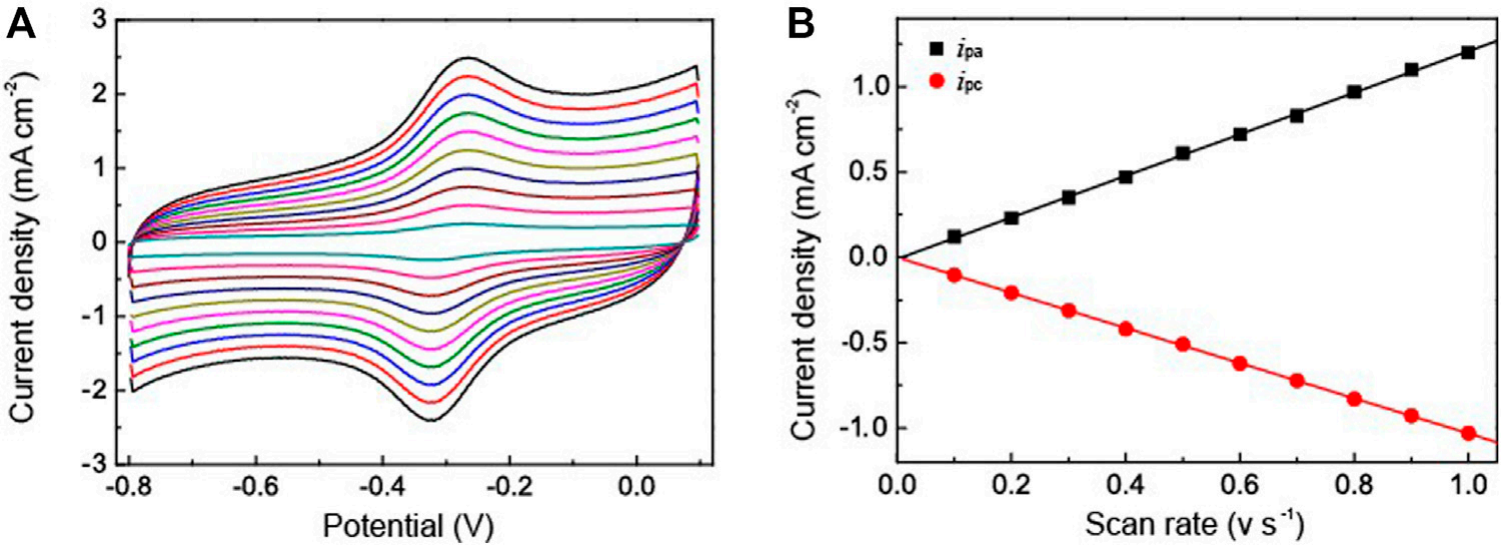
**(A)** CV curves of HRP/Au NPs-PDA-PAA-graphene at various scan rate ranged from 0.1—1V/s in PBS (pH 7.4) **(B)** is the plot of cathodic/anodic peak current vs. scan rate.

To further clarify the catalytic activity between the HRP and the electrode we used the Au NPs-PDA-PAA-graphene modified electrode immobilized with HRP to study the thoroughgoing current response by applying a different scan rate. As represented in Figure 5A, although the redox current gradually increased with an increase in the scan rate, the peak potential did not change obviously. Figure 5B shows the excellent linear relation between the redox peak current values and increased scanning rate, ranged from 0.1—1 V/s. Here the regression equation;

$$i_{pa}=1.00\times 10^{-4}+0.122\text{v}\left(\text{R}=0.999\right)$$

$$i_{pc}=-9.33\times 10^{-5}-0.103\text{v}\left(\text{R}=0.999\right)$$

Here, v represents the scan rate while *i*_*pa,*_ *i*_*pc*_ are the peak current density of anode and cathode. This suggests that the redox reaction of HRP is a typical facet constrained reversible phenomenon. With the assimilation of current (reduction peak) and Faraday’s law, the electrochemically active HRP can be calculated from HRP/Au NPs-PDA-PAA-graphene surface from the equation. (Zhang et al., 2010),

$$i_{pa}=\frac{n^{2}F^{2}A\Gamma \nu }{4RT}$$

where ipa signifies the reduction peak current, n denotes the total amount of electrons, A stands for the electrode surface area, v is the scan rate, and Γ is the value which needs to be calculated. The estimated Γ value was 9.57 × 10^−11^ mol cm^−2^. The Au NPs-PDA-PAA-graphene composite matrix gives remarkable higher surface concentration value for the immobilization of HRP as compared to the previously published literature using different types of matrix, such as colloidal Au (7.5 × 10^–11^ mol cm^−2^) (Liu and Ju, 2002)^]^, 3-mercaptopropionic acid monolayer-modified gold surface (5 × 10^−11^ mol cm^−2^) (Li and Dong, 1997).

**FIGURE 6:**
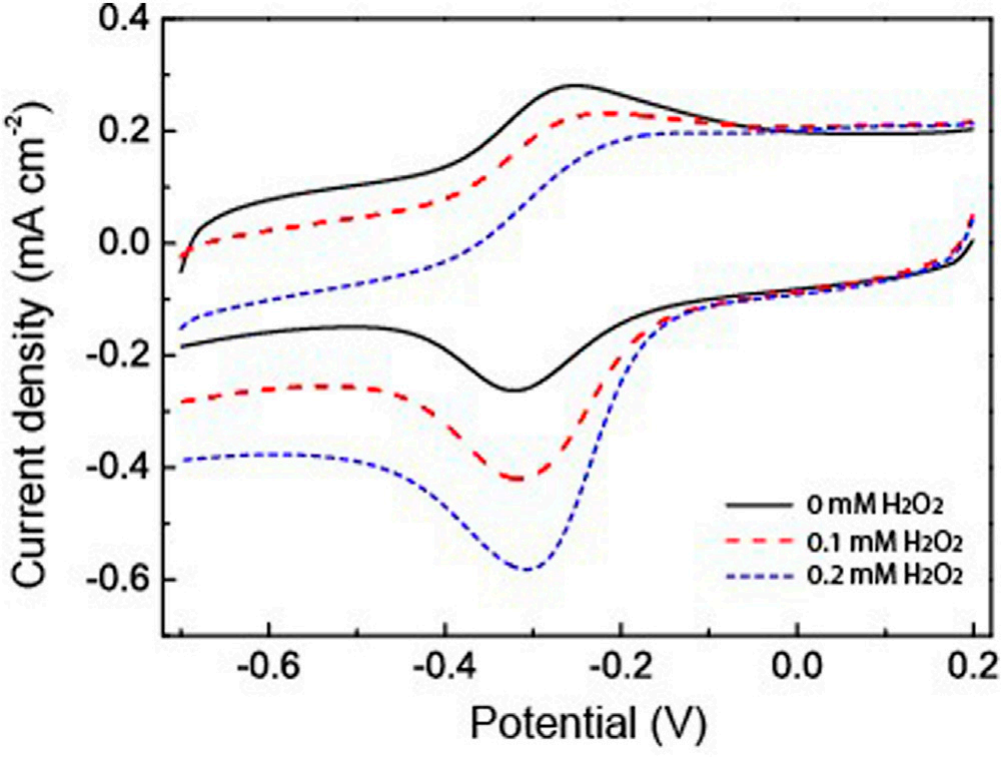
CV curves of HRP/Au NPs-PDA-PAA-graphene with and without H_2_O_2_ in PBS (pH 7.4). Scan rate: 50 mV/s.

H_2_O_2_ has been used as an analyte to study the catalytic applications of the fabricated HRP/Au NPs-PDA-PAA-graphene films. Figure 6 explained the catalytic behavior of the fabricated material in the presence and absence of H_2_O_2_. When the different concentrations of H_2_O_2_ were injected into PBS, there was a clear rise in the reduction peak current with a gradual decrease in the oxidation peak current. This phenomenon well explains the remarkable electrocatalytic process for the HRP oxidation *via* H_2_O_2_ which in turn was reduced. H_2_O_2_ oxidized the HRP_re_ to HRP_ox_ which was further reduced through electron transfer upon the electrode surface. As H_2_O_2_ concentration increased it encouraged a large cathodic peak current value, in turn there was a fast rate of electron transfer between the quantities of electrode surface and immobilized HRP.

**FIGURE 7:**
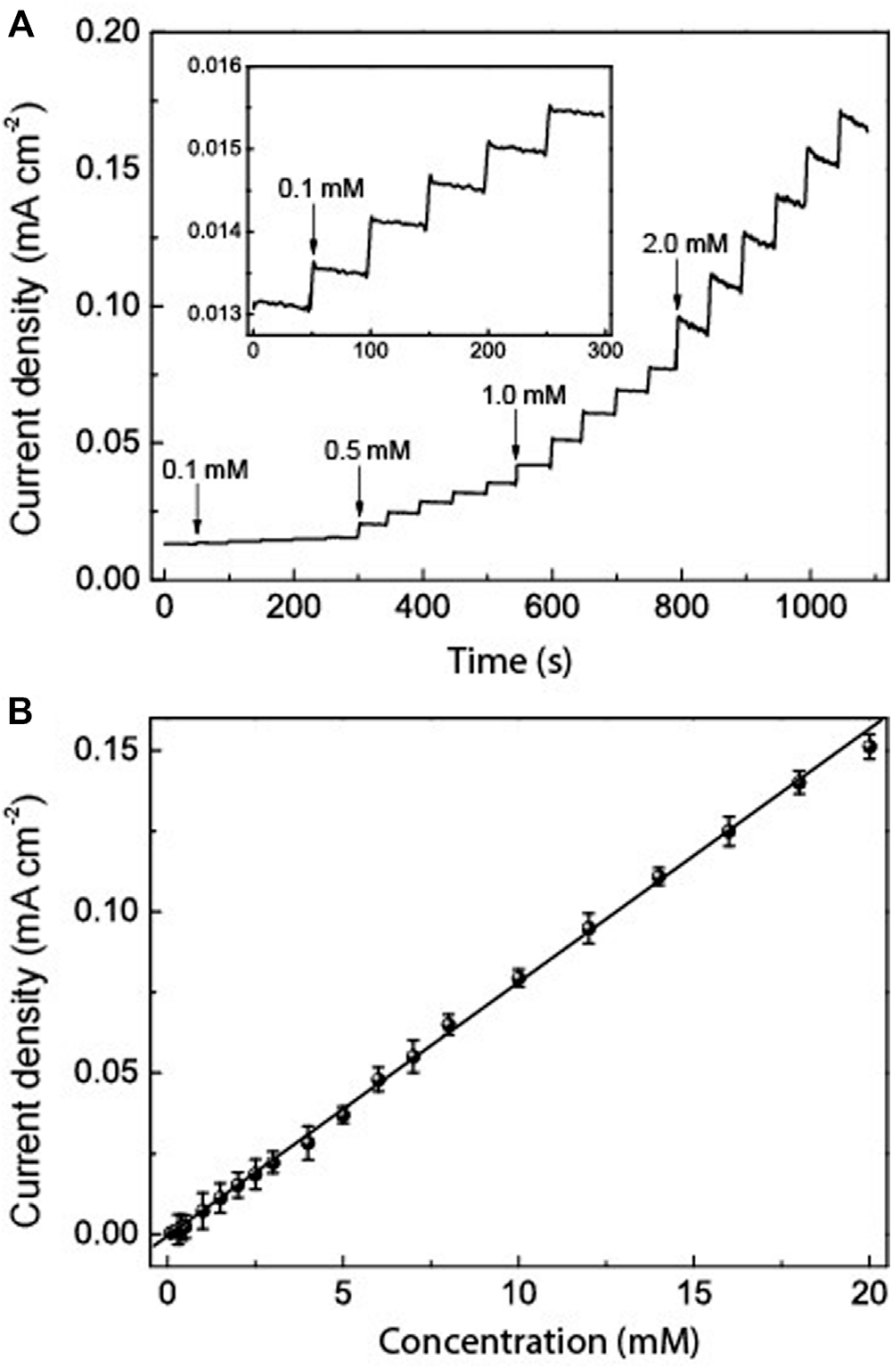
**(A)** Typical amperometric response of HRP/Au NPs-PDA-PAA-graphene film electrode after the sequential adding various concentrations of H_2_O_2_. The inset is the enlarged response with the lowest concentration of H_2_O_2_ **(B)** The linear calibration curves of HRP/Au NPs-PDA-PAA-graphene film electrode for H_2_O_2_ determination.

The amperometric response of the HRP/Au NPs-PDA-PAA-graphene film electrode with sequential injection of H_2_O_2_ in PBS at −0.32 V is shown in Figure 7. The fabricated electrode gives a quick response towards any change in the H_2_O_2_ concentration and attains a steady state within 5 s, which is credited to the rise in electron transfer from the embedded Au NPs. The lowest limit of detection (0.02 μM S/N = 3) and an excellent linearity range from 0.1 μm to 20 mm (R = 0.999) is better than that in similar systems previously reported (Lin et al., 2007; Lu et al., 2011; Liu et al., 2012). These results suggest that HRP/Au NPs-PDA-PAA-graphene nanoarchitectures pave the way for the synthesis of enzymatic biosensors for the sensitive detection of H_2_O_2._

The repeatability parameter was evaluated by taking 10 repeated measurements with the consecutive amount of 1.0 mm H_2_O_2_ with a same electrode which produces relative standard deviation (RSD) of ∼3%. Furthermore, the RSD for the current responses for more than 10 sensors obtained by following the similar protocol is ∼5%. For the stability evaluation of the HRP/Au NPs-DA-PAA-graphene film electrode preserved at 4°C, it gives 90% response even after 2 months of refrigeration (Supplementary Figure S3). The application of Au NPs-PDA-PAA-graphene composite enhances the biocompatibility and provides a favorable microenvironment for HRP, which aids the retention of the biological activity of HRP. Meanwhile, we speculate that the hydrophilic character of Au NPs-PDA-PAA-graphene would also improve the HRP catalytic performance it does not gets denaturation as well as no reduction in catalytic functioning has been observed at low temperature. Conversely, the sensor sensitivity get reduced quickly at room temperature which may be the reason for the swift deactivation of HRP.

**FIGURE 8:**
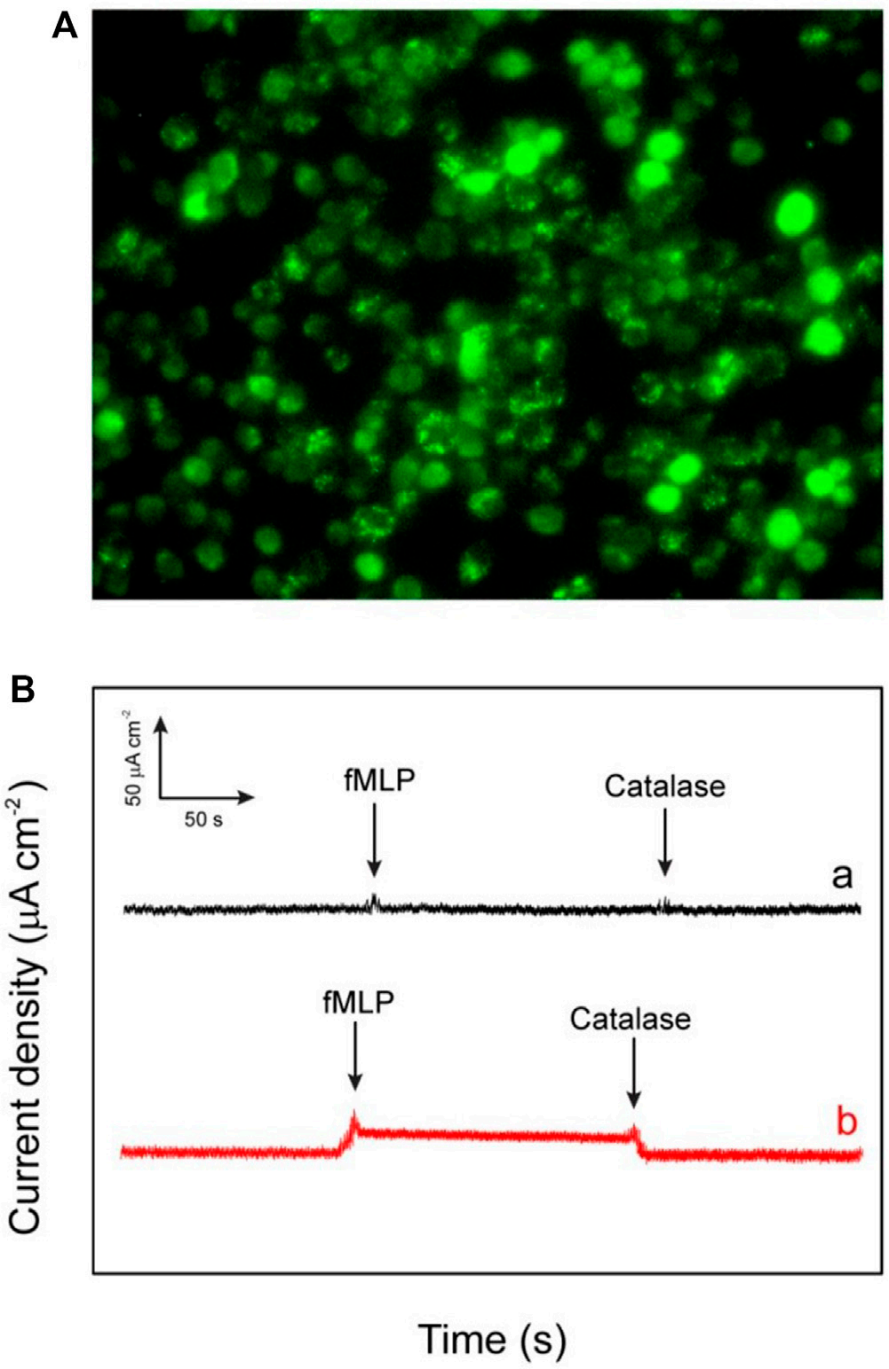
**(A)** The dark field image of the macrophages (RAW264.7 murine) investigated for the *in vitro* applications **(B)** Amperometric response of HRP/Au NPs-PDA-PAA-graphene electrode for measuring the reducibility of H_2_O_2_ released macrophage cells induced upon the addition of 0.3 mm fMLP **(A)** and without addition of 50 ml of catalase (300 UmL^−1^) **(B)**. The electrode was checked at bias value of a consistent potential of −320 mV (versus SCE).

By keeping in mind all the advantages of the excellent catalytic performances of the fabricated electrode such as lowest limit of detection, enhanced sensitivity, and quick response, it is noteworthy to say that the as-fabricated biosensor can be used for the determination of the extracellular H_2_O_2_ released by macrophage cells. The used macrophages offer a pivotal role against the different kind of microorganisms and tumors in there host cells (Niedel et al., 1979). However, these two special properties (tumoricidal and microbicidal) of the chemotactically responsive macrophages required some types of agents like bacterial products, cytokines, synthetic peptide, and some chemotherapeutic drugs for their activation. The stimulator N-formyl-methionyl-leucyl-phenylalanine (fMLP) used in this work is a synthetic peptide which is structurally identical to oligopeptide products of bacterial metabolism (Holian and Daniele, 1981). The chemistry behind the use of fMLP as a stimulator is not only its chemotactic properties but it also facilitates the adhering of PMN to endothelium cells. Moreover it also stimulates the release of many kind of molecules such as reactive oxygen intermediates (ROI), lysosomal enzymes, nitric oxide, interleukin 1 (IL-1), and tumor necrosis factor. In this work, the cell macrophages regarding 80% confluency are subjected to release H_2_O_2_ after injecting fMLP. In the experiment of real-time sensing of H_2_O_2_ secretion by macrophages, calcein-AM is used to stain the live cells. The dark field microscopic images refer to the fact that the stained macrophages cells are well-spread and healthy (Figure 8A). According to the experimental results, after the addition of each 10 μm concentration of fMLP a clear current increase (2.8 μA cm^−2^) has been observed on the HRP/Au NPs-PDA-PAA-graphene film electrode at the applied potential (−0.32 V), which then further decreases as the H_2_O_2_ scavenger catalase was spiked. The same procedure was carried out with control well plates (without macrophages) which showed no response as depicted by Figure 8B). Therefore, we can conclude that the reported rise in cathode current at HRP/Au NPs-PDA-PAA-graphene film electrode placed near the cells is endorsed to H_2_O_2_oxidation. All the above discussed results markedly validate that the as-fabricated biosensor can initiate a new opening in biosensing platforms for the unswerving determination of extracellular as well as intracellular H_2_O_2_ and could well be effectively helpful for advanced physiological and pathological related studies.

## Conclusion

In summary, new Au NPs-PDA-PAA-graphene composite films have been synthesized by loading highly active enzyme immobilization. The material is introduced onto the electrode surface, and serves as biosensor for extracellular and intracellular H_2_O_2_ detection. The biosensor exhibits remarkable results in terms of extraordinary sensitivity, wider linear range (0.1 μm–20 mm), noble stability, lowest limit of detection (0.02 mm), and reproducibility for H_2_O_2_ detection, which is superior to previously constructed biosensing podiums. Hence, this work brings up an advanced biosensing platform for the determination of H_2_O_2_ *in vivo* and *in vitro*, which can be of utmost advantage for bioelectroanalytical chemistry, cellular biology, and pathophysiological studies.

## Data Availability

The original contributions presented in the study are included in the article/Supplementary Material, further inquiries can be directed to the corresponding author.
