# Supplementary material for: A Multicomponent Polymer-Metal-Enzyme System as Electrochemical Biosensor for H2O2 Detection
Source: Front Chem. 2022 Apr 29;10:874965. doi: 10.3389/fchem.2022.874965 (PMC9099068; doi:10.3389/fchem.2022.874965)
Supplement: Supplementary file 1 [file DataSheet1.docx]

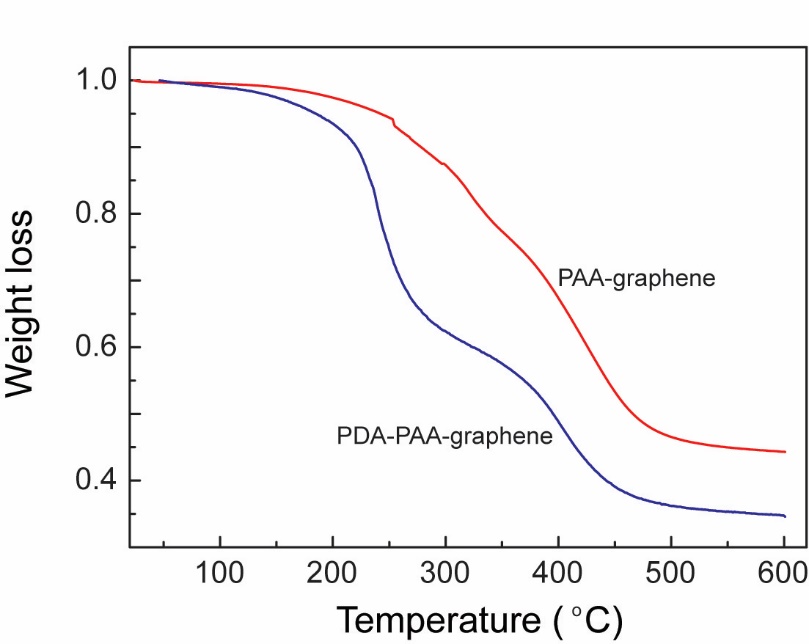


**Supplementary Figure 1.** TGA for PAA-graphene and PDA-PAA-graphene.
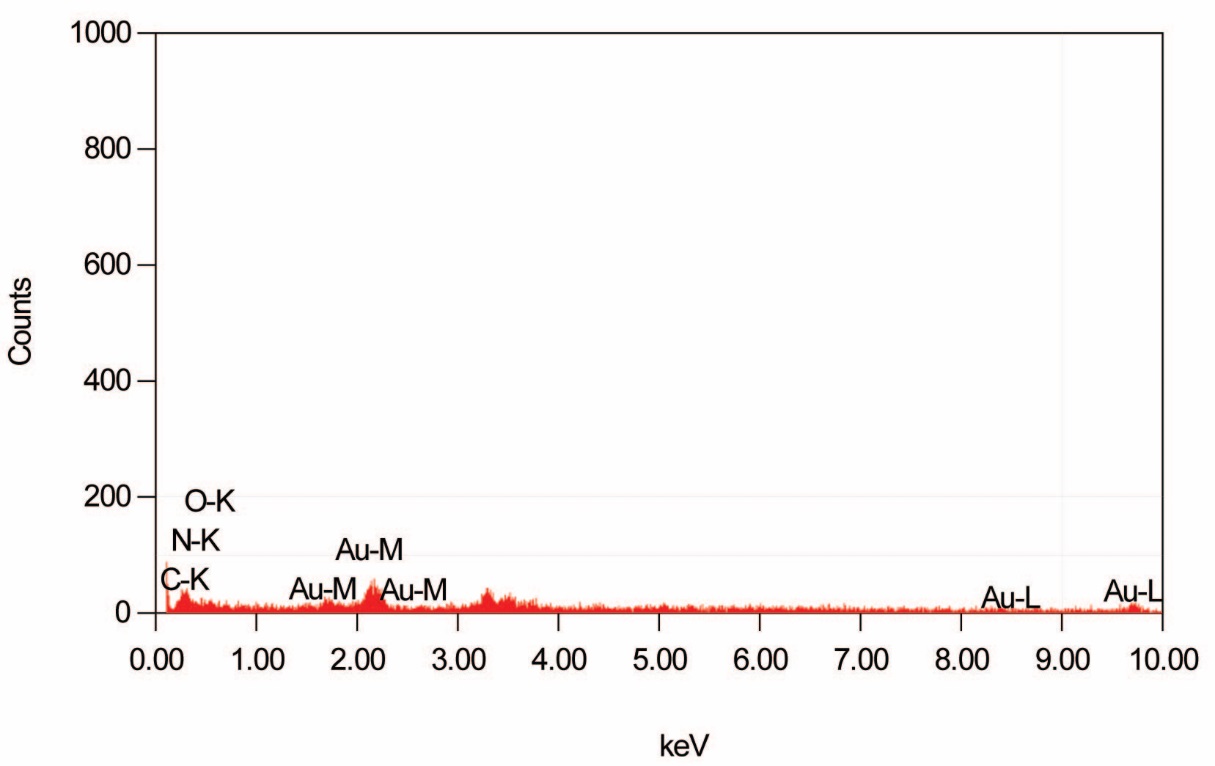


**Supplementary Figure 2.** EDS spectrum of AuNPs-PDA-PAA-graphene.


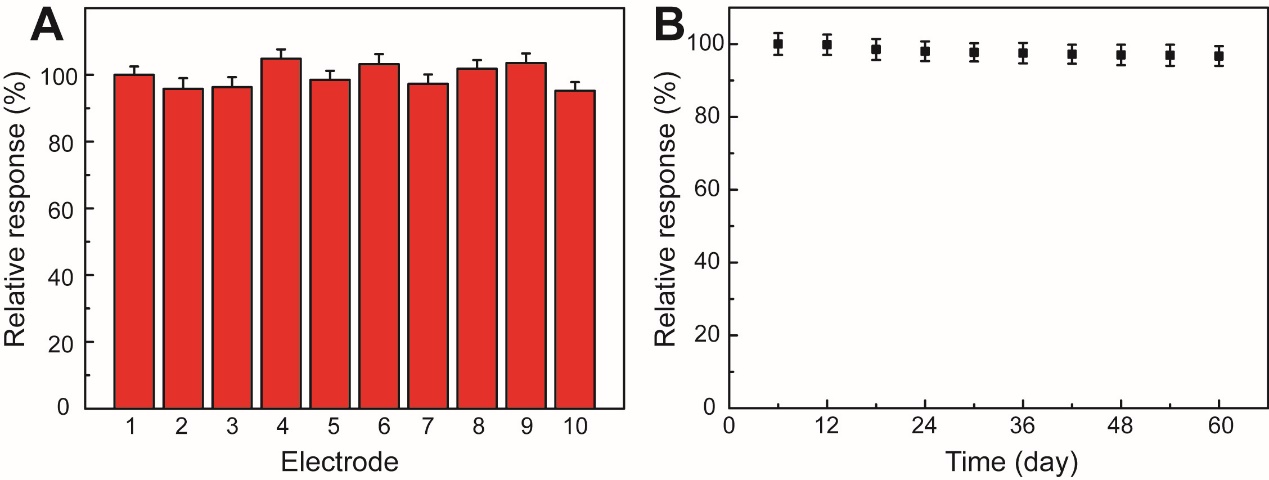


**Supplementary Figure 1.** (A) Variation of the response of 10 different AuNPs-PDA-PAA-graphene electrodes to 1.0 mM H_2_O_2_. (B) The current response of 10 different AuNPs-PDA-PAA-graphene electrodes to 1.0 mM H_2_O_2_ with time.
